# Monthly Follow-Ups of Functional Status in People with COPD: A Longitudinal Study

**DOI:** 10.3390/jcm11113052

**Published:** 2022-05-28

**Authors:** Vânia Rocha, Jorge Cabral, Sara Souto-Miranda, Ana Filipa Machado, Cristina Jácome, Joana Cruz, Vitória Martins, Paula Simão, Maria Aurora Mendes, Vera Afreixo, Alda Marques

**Affiliations:** 1 Respiratory Research and Rehabilitation Laboratory (Lab3R), School of Health Sciences (ESSUA), University of Aveiro, 3810-193 Aveiro, Portugal; vania.rocha@ua.pt (V.R.); sara.souto@ua.pt (S.S.-M.); filipamachado@ua.pt (A.F.M.); joana.cruz@ipleiria.pt (J.C.); mamendes88@gmail.com (M.A.M.); 2 Institute of Biomedicine (iBiMED), University of Aveiro, 3810-193 Aveiro, Portugal; 3 Center for Research & Development in Mathematics and Applications (CIDMA), Department of Mathematics, University of Aveiro, 3810-193 Aveiro, Portugal; jorgecabral@ua.pt (J.C.); vera@ua.pt (V.A.); 4 Center for Health Technology and Services Research (CINTESIS), Faculty of Medicine, University of Porto (FMUP), 4200-450 Porto, Portugal; cjacome@med.up.pt; 5 Department of Community Medicine, Information and Health Decision Sciences (MEDCIDS), Faculty of Medicine, University of Porto (FMUP), 4200-450 Porto, Portugal; 6 ciTechCare-Center for Innovative Care and Health Technology, School of Health Sciences (ESSLei), Polytechnic of Leiria, 2411-901 Leiria, Portugal; 7 Pulmonology Department, Hospital Distrital da Figueira da Foz, 3094-001 Figueira da Foz, Portugal; vitoria.b.martins@gmail.com; 8 Pulmonology Department, Unidade Local de Saúde de Matosinhos, 4450-021 Matosinhos, Portugal; simao.paula@gmail.com; 9 Pulmonology Department, Centro Hospitalar do Baixo Vouga (CHBV), 3810-096 Aveiro, Portugal

**Keywords:** COPD, functional status, evaluation and monitoring, field tests, one-minute sit-to-stand test

## Abstract

Functional status is an important and meaningful outcome in people with chronic obstructive pulmonary disease (COPD), although its measurement is not embedded in routine clinical assessments. This study described the functional status of people with COPD using the 1-min sit-to-stand test (1minSTS) over a 6-month period and the examined sociodemographic and clinical characteristics associated with this outcome. Data from a prospective study including people with COPD were analyzed. Functional status was assessed monthly with the 1minSTS over 6 months. Linear-mixed effect models assessed the 1minSTS number of repetitions mean change. One-hundred and eight participants (82.4% men; 66.9 ± 9.5 years) were included. A significantly lower number of repetitions in the 1minSTS over the 6-month period was associated with being female (estimate: −4.69, 95%CI: −8.20; −1.18), being older (estimate: −0.56, 95%CI: −0.77; −0.34), having higher BMI (estimate: −0.55, 95%CI: −0.81; −0.28) and having higher activity-related dyspnea (estimate: −2.04, 95%CI: −3.25; −0.83). Half of the participants showed improvements above three repetitions in the 1minSTS over the 6-month period, independently of their baseline impairment (1minSTS < 70% predicted: 52.5%; ≥70% predicted: 54.4%). To conclude, monthly follow-up assessments were associated with clinically relevant benefits in the functional status of people with COPD. Age, body composition, and activity-related dyspnea were the main predictors of functional status over time. Further research is needed to corroborate our findings and to support the beneficial effects of regular COPD monitoring.

## 1. Introduction

Chronic obstructive pulmonary disease (COPD) is the third leading cause of mortality worldwide [1]. This condition is characterized by airflow limitation and persistent and progressive pulmonary and extra-pulmonary manifestations [2], which lead to functional status decline.

Functional status is an important and meaningful outcome in people with COPD [3]. It refers to the ability to provide for life’s necessities, i.e., activities that people do during their lives to meet basic needs, fulfil usual roles, and maintain their health and wellbeing [4,5]. Prior evidence has shown that poor functional status is a predictor of acute exacerbations, hospital admissions, and increased mortality in people with COPD [6,7].

A number of tests have been proposed to assess functionality [8,9,10], and the six-minute walking test (6MWT) has been the most used [11,12]. The one-minute sit-to-stand test (1minSTS) also shows potential to measure functional status, namely in space-limited places (e.g., patients’ home, physiotherapy clinics), being useful across different settings [13,14]. Furthermore, it is a simple, objective, valid, and responsive test that mimics a meaningful activity of daily living (sitting and standing from a chair), considered essential to maintain independence in people with COPD [13]. However, measurement of functional status is still not embedded in clinical practice, and its routine assessment using a minimal resource test as the 1minSTS remains poorly explored [10].

Therefore, this study aimed to describe the functional status of people with COPD using the 1minSTS over a 6-month period and to examine the sociodemographic and clinical characteristics associated with this outcome.

## 2. Materials and Methods

### 2.1. Study Design and Participants

This study was an observational longitudinal study, part of a prospective study (PRIME-PTDC/SAU-SER/28806/2017 (ClinicalTrials.gov Identifier: NCT03701945) aiming to establish the effects of community-based pulmonary rehabilitation in people with COPD. It also aimed to describe the clinical trajectory of the disease over 6 months, independently of patients’ participation in pulmonary rehabilitation programmes. The study was conducted between November 2018 and August 2020. The Ethics Committees of Unidade Local de Saúde de Matosinhos (ref. 10/CES/JAS 17 February 2017 and 73/CE/JAS 12 October 2018), Centro Hospitalar Baixo Vouga (ref. 777638 and 086892), Hospital Distrital da Figueira da Foz (ref. 1807/2017 and 27 May 2019), and Administração Regional de Saúde do Centro (ref. 64/2016 and 85/2018) approved the study. This study is reported according to the Strengthening the Reporting of Observational Studies in Epidemiology (STROBE) guidelines [15].

People with COPD living in the community were recruited by physicians at three hospitals during their routine pulmonology appointments. Individuals were eligible for this study if diagnosed with COPD [2], clinically stable for 1 month prior to the study (i.e., no hospital admissions or exacerbations nor changes in medication [2]) and did not undergo pulmonary rehabilitation during the study period or 6 months before the baseline assessment. We decided to exclude participants who participated in pulmonary rehabilitation due to the demonstrated benefits of this intervention in functional status [16,17]. Exclusion criteria included the presence of other respiratory diseases or significant cardiovascular, neurologic, or musculoskeletal disease that precluded their participation in the study. Written and verbal descriptions of the study were provided to eligible participants. Those interested were invited to have their health status monitored monthly over a 6-month period. Written informed consent was obtained from all participants before data collection.

### 2.2. Data Collection

Sociodemographic (age, sex, and educational level) and anthropometric (height and weight to compute body mass index (BMI)) data were first collected using a structured questionnaire. Participants’ educational level was measured as completed years of schooling and classified into three categories according to the International Standard Classification of Education (ISCED) [18]: ≤4 years (ISCED 0–1); 5–9 years (ISCED 2); ≥10 years (ISCED 3–8), following the current Portuguese education system. Clinical data were also collected and included smoking habits, activity-related dyspnoea, impact of the disease, number of hospitalizations and acute exacerbations of COPD (AECOPD) in the preceding year, comorbidities, and medication. Lung function was obtained from participants’ medical records and used to establish the severity of airway obstruction according to GOLD [2].

Smoking status was stratified into never, former, or current smoker, and pack-years were calculated [19]. Activity-related dyspnoea was assessed with the modified British medical research council dyspnoea (mMRC) scale, which ranges from 0 (no trouble with breathlessness) to 4 (too breathless to leave the house) [20]. The COPD assessment test (CAT) [21,22] was used to evaluate the impact of the disease and to classify participants in GOLD groups according to the ABCD assessment tool [2]. The severity of comorbid diseases was scored using the Charlson Comorbidity Index (CCI) (i.e., mild: score 1–2; moderate: score 3–4; severe: score ≥5) [23]. Information on the use of long-term oxygen therapy and non-invasive ventilation was also registered.

Functional status was assessed with the 1minSTS at baseline and monthly, up to six months. The 1minSTS is a simple and quick test, which requires less than 2 squared-meters of space to be performed [13]. It consists of sitting and standing from a 46–48 cm height chair as many times as possible, at a self-selected pace, without using arms as support, for one minute [14]. Individuals may use rest periods to complete the 1-min period [14]. The number of completed repetitions was recorded and expressed as absolute values. Percentage predicted values were calculated based on available reference values [24]. The minimum clinically important difference (MCID) of 3 repetitions was used as a reference for a clinically relevant improvement [14].

### 2.3. Data Analysis

Descriptive statistics were used to characterise the sample. Continuous variables were summarised as mean and standard deviation or median and first and third quartiles (Q), while count variables were summarised as numbers and proportions. A box plot was computed to describe the number of repetitions in the 1minSTS over the 6-month period. Linear-mixed effect models with random intercepts and slopes were applied to assess the mean change in number of repetitions [25,26]. A backward elimination with single term deletion was performed, and the model with the lowest Akaike information criterion (AIC) value was chosen [27]. Intraclass correlation coefficient (ICC), marginal coefficient of determination (marginal R2), and conditional coefficient of determination (conditional R2) were determined to assess the model’s quality and level of adjustment [28]. 

Differences in the number of repetitions of the 1minSTS between the baseline and the 6th evaluation were assessed with the Wilcoxon signed-rank test and were used to interpret the MCID. These differences were calculated for participants with or without baseline impairment, i.e., 1minSTS <70% predicted or 1minSTS ≥70% predicted [24]. A complete case analysis was considered, and only participants with complete information in the 1minSTS as well as sociodemographic and clinical data over the 6-month period were included. Two-sided *p*-value < 0.05 was considered statistically significant. Statistical analyses were performed using R packages joinerR, nlme, and ggeffects in RStudio version 2022.2.0.443 running R version 4.1.2 [29,30,31,32].

## 3. Results

A total of 108 participants (from the 179 participants who accepted to participate in the study) were included. Reasons for exclusion were incomplete information in the variables of interest (n = 30) or being involved in a pulmonary rehabilitation program during the follow-up period or 6 months before the baseline (n = 41). No significant differences were observed in sociodemographic characteristics, lung function, GOLD groups, CCI score, and 1minSTS between included and excluded participants, except in sex, with more males in the included than excluded participant groups (84% vs. 50%). Participants’ mean age was 67 (±10) years old, with a median FEV_1_ % predicted of 58 [41;75], and most were men (82%). Further baseline characteristics are described in Table 1.

**Figure 1:**
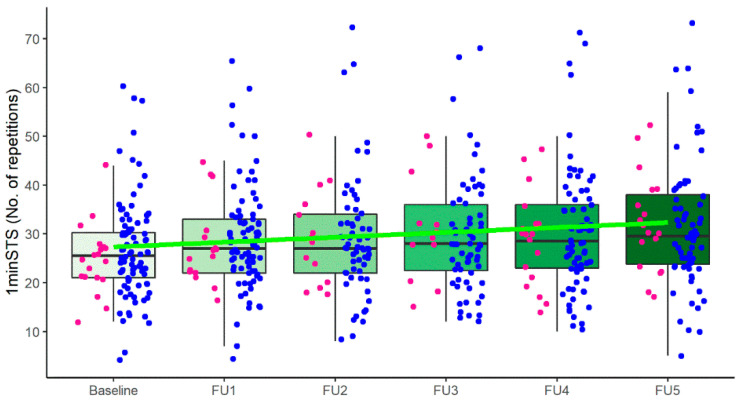
Number of repetitions in the one-minute sit-to-stand test (1minSTS) over 6 months. Time was defined as number of days between baseline and follow-up assessments; the green line represents the linear tendency, considering time a continuous variable; blue dots represent male patients and pink dots represent female patients; No.: number.

A significant increase in the median number of repetitions of the 1minSTS from 25.5 (21.0; 30.3) at baseline to 30.0 (24.0; 37.5) at month six (*p* < 0.001) was observed (Figure 1).

**Figure 2:**
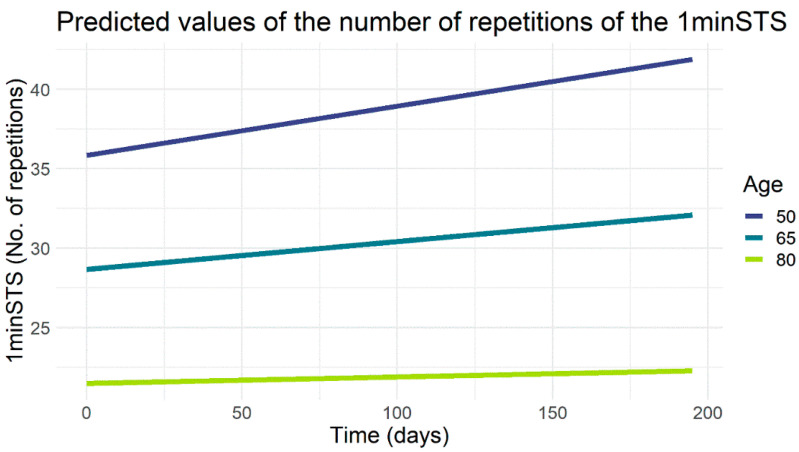
Predicted values for the number of repetitions in the one-minute sit-to-stand test (1minSTS) in participants with chronic obstructive pulmonary disease. According to the model, the 1minSTS values were adjusted for male participants, body mass index of 25, mild Charlson comorbidity index score, 53.31% predicted of forced expiratory volume in one-second, and a score of 2 in the modified British medical research council dyspnoea scale.

Table 2 shows the results of the linear mixed-effects models, considering time as a continuous variable and participants as random effects. A substantial explanatory power of the 1minSTS model (conditional R2: 0.92) was observed. A significantly lower number of repetitions in the 1minSTS was associated with female sex (estimate: −4.19, 95%CI: −8.15; −0.23), older age (estimate: −0.48, 95%CI: −0.64; −0.32), higher BMI (estimate: −0.51, 95%CI: −0.84; −0.17), and higher mMRC scores (estimate: −2.89, 95%CI: −4.24; −1.55). Statistically significant interactions between age and time (estimate: −8.89 × 10^−4^, 95%CI: −1.61 × 10^−3^; −1.86 × 10^−4^) were observed (Table 2). Specifically, the model predicted significantly lower values in the 1minSTS number of repetitions and a less pronounced increase over time in older participants compared to younger participants (Figure 2). Thus, the 1minSTS number of repetitions at 50, 65, and 80 years of age were 35.8, 28.7, and 21.5 at baseline and increased to 41.9, 32.1, and 22.3 at month six, respectively (Figure 2). 

**Figure 3:**
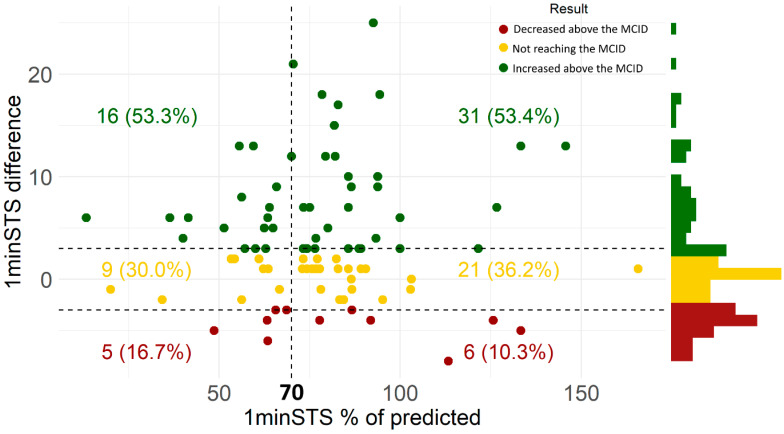
Change in the one-minute sit-to-stand (1minSTS) number of repetitions from the first to the sixth assessment in participants with baseline functional status impairment (i.e., 1minSTS < 70% predicted—represented as dots in the left side of the vertical dashed line) or without impairment (i.e., 1minSTS ≥ 70% predicted—represented as dots in the right side of the vertical dashed line).The vertical dashed line illustrates the established impairment cut-off; horizontal dashed lines illustrate the minimal clinically important differences interval (MCID): ±3 repetitions; green dots represent participants who had their number of repetitions increased above the MCID; yellow dots represent participants who had their number of repetitions similar or unchanged with time without reaching the MCID; red dots represent participants who had their number of repetitions decreased above the MCID.

Figure 3 illustrates the change in the one-minute sit-to-stand (1minSTS) number of repetitions from the first to the sixth assessment in participants with (<70% of predicted) or without (>70% of predicted) baseline functional status impairment. Half of participants showed improvements ≥3 repetitions (MCID) in the 1minSTS over the 6-month period follow-up, independently of the baseline impairment (<70% predicted −53.3%; ≥70% predicted −53.4%) (Figure 3). According to the model, a significant increase of 4.2 repetitions in the 1minSTS is expected after the 6-month period with monthly routine assessments (Figure S1).

## 4. Discussion

This study showed that monthly follow-up assessment of people with COPD was associated with clinically relevant benefits in their functional status. Specifically, an increase of four repetitions in the 1minSTS was observed, surpassing the established MCID of three repetitions [14], even in participants with no baseline impairment in functional status.

Previous studies [33,34,35,36] suggested that regular monitoring is important to assess physical activity and improve overall health status, health-related quality of life, and disease prognosis of people with COPD, yet this was the first study to describe the effects of continuous monitoring in their functional status using the easy and quick-to-apply measure of the 1minSTS, not associated with pulmonary rehabilitation [37] or maintenance exercise programmes [38]. Although recommendations indicate that people with COPD should be monitored annually or biannually, depending on the disease severity [2,39], evidence has started to emerge showing the clinical importance of more frequent monitoring of this population to increase their quality of life, reduce exacerbations, improve personalised care, and reduce costs [6,7,36]. Thus, this study extends findings from earlier research by showing that an increase in the frequency of monitoring or a close monitoring (e.g., domiciliary visits) might be associated with improvements in the functional status of people with COPD and deserve to be further explored.

We also found functional status to be lower in females, older people, and people with higher BMI and higher levels of activity-related dyspnoea. While sex and age constitute non-modifiable factors, body composition (i.e., overweight/obesity) and activity-related dyspnoea are important preventable and modifiable factors that can be addressed in interventions for people with COPD. In fact, comprehensive interventions, such as pulmonary rehabilitation, that include multiple therapies (e.g., exercise training, nutritional education, psychosocial support) [40] and/or maintenance exercise programmes have clearly demonstrated benefits and should therefore be part of the integrated care provided to this population [41,42].

The results of our predictive models showed that increased age was the main predictor of functional status decline, confirming previous studies [43,44]. Nevertheless, our findings also suggest that people with COPD might improve their functional status regardless of age (Figure 2), and even participants without impairment in this domain are able to improve (Figure 3). Thus, we may hypothesize that an adequate follow-up of people with COPD using minimal resources might improve the functional status of people with COPD in all age groups, and therefore investments to incorporate this health domain in routine clinical assessments and treatment are worthwhile. On the other hand, we found that some participants did not improve their functional status over time, independently of relevant clinical factors such as exacerbations, COPD symptoms, health-related quality of life, and other clinical variables. These variables were therefore included in the model and demonstrated to be not statistically associated to participants’ functional status over time. A prior study [17] also demonstrated that about half of people with COPD were found to be non-responders, even when included in pulmonary rehabilitation, highlighting the need for further investigation exploring the reasons/predictors of treatment response in people with COPD.

Our findings are also challenging to explain, since continuous monitoring showed improvements above the established MCID of three repetitions for pulmonary rehabilitation [14]. The 1minSTS has shown to be less prone to the learning effect than, for example, the 6MWT; therefore, other factors should be considered as possible mechanisms explaining this improvement [14,16]. For instance, the benefits of the regular contact with healthcare professionals (e.g., feeling of support, responsibility to follow the plan, the unplanned advice provided by these professionals on the importance of being physically active) have been reported and offer a possible explanation for this improvement [2,45,46]. More studies are needed to disentangle the factors inherent to functional status improvement in people with COPD.

### Strengths and Limitations

The longitudinal design, including an objective assessment of functional status with a simple and feasible measure for people with COPD, is the main strength of this study. Some limitations should also be considered. Firstly, the follow-up period of 6 months may limit our conclusions, since we were not able to test if the beneficial effects associated with the monthly monitoring would be maintained over a longer period. A higher sample size would strengthen our results. However, in repeated measures studies, a sample higher than 100 individuals is considered adequate [47], and our study includes more than 500 observations. Moreover, the COVID-19 pandemic limited data collection, since fewer domiciliary visits were performed, and we had to stop data collection before the end of the project to avoid the potential risk of infection for people with COPD. A high variability in the number of repetitions in the 1minSTS was also found, namely at the end of the follow-up, which might be explained by the large age range and COPD grades included in our study. In fact, individuals with the highest performance were younger and mostly from COPD grades A and B. Previous research [16,17] also demonstrated negative correlations of this test with the COPD assessment test CAT (used to classify COPD grades) and the number of repetitions was higher in younger populations [16]. Moreover, a causal relationship could not be established, since we could not control for all possible factors explaining this improvement. For instance, it was not possible to study in detail the physical activity levels, type of comorbidities, and medication, which might have potential effects in functional status [4,48]. Therefore, more research is needed to corroborate our findings and establish an adequate frequency for clinical monitoring of people with COPD [49]. Finally, the sociodemographic characteristics of our sample (i.e., older adults from 59 to 77 years old, mainly male) might influence external validity to all people with COPD, although these characteristics correspond to the usual characteristics of people with COPD.

## 5. Conclusions

This study found clinically relevant benefits in the functional status of people with COPD during monthly follow-up assessments. Age, body composition, and activity-related dyspnoea were the main predictors of functional status over time; thus, interventions should consider these factors when providing integrated care to this population. Further research is needed to corroborate our findings and to establish the frequency and beneficial effects of monitoring people with COPD as a possible way to optimise disease management.

## Figures and Tables

**Table 1:** Baseline characteristics of participants with chronic obstructive pulmonary disease (n = 108).

Characteristics (n = 108)	
**Age**, years—mean (SD)	66.9 (9.5)
**Male**	89 (82.4)
**BMI**, kg/m^2^—mean (SD)	26.9 (4.4)
**Educational level**, years	
≤4 (ISCED 0–1)	52 (48.1)
5–9 (ISCED 2)	29 (26.9)
≥10 (ISCED 3–8)	27 (25.0)
**Smoking status**	
Never	13 (12.0)
Former	81 (75.0)
Current	14 (13.0)
**Pack-years**—median (Q1–Q3)	45.0 (17.7–75.9)
**mMRC**—median (IQR)	1 (1.0; 2.0)
**AECOPD**, in previous year	
0	79 (73.1)
1	10 (9.3)
>1	19 (17.6)
**Lung function**	
FEV_1_, % predicted—median (Q1–Q3)	58.0 (41.0–75.0)
FEV_1_/FVC—median (Q1–Q3)	54.7 (45.3–63.0)
GOLD grades	
1	23 (21.3)
2	44 (40.7)
3	27 (25.0)
4	13 (12.0)
**CAT**, total score—median (Q1–Q3)	11.0 (8.0–18.0)
**Long-term oxygen therapy**	16 (15.0)
**Non-invasive ventilation**	18 (16.7)
**GOLD groups**	
A	39 (36.1)
B	47 (43.5)
C	1 (0.9)
D	21 (19.4)
**CCI score**	
Mild (1–2 points)	25 (23.1)
Moderate (3–4 points)	58 (53.8)
Severe (≥5 points)	25 (23.1)
**Medication**	
Bronchodilators	
ICS	11 (10.2)
LAMA	23 (21.3)
SABA	16 (14.8)
LABA	10 (9.3)
SAMA	1 (0.9)
Combination ICS/LABA	31 (28.7)
Combination LABA/LAMA	34 (31.5)
Combination LABA/LAMA/ICS	2 (1.9)
Xanthines	11 (10.2)
Expectorants	6 (5.6)
LTRA	5 (4.6)
Anti-fibrotics/ Immunosuppressants	1 (0.9)
**1minSTS**, number of repetitions—median (Q1–Q3)	25.5 (21.0–30.3)
<70% predicted	45 (41.7)
≥70% predicted	63 (58.3)

**Legend:** Data are presented as n (%), unless otherwise stated. 1minSTS, one-minute sit-to-stand test; AECOPD, acute exacerbation of COPD; BMI, body mass index; CAT, COPD assessment test; CCI, Charlson comorbidity index; COPD, chronic obstructive pulmonary disease; FEV_1_, forced expiratory volume in 1 s; FVC, forced vital capacity; GOLD, global initiative for chronic obstructive lung disease; ICS, inhaled corticosteroids; ISCED, International Standard Classification of Education; LABA, long-acting beta-agonist; LAMA, long-acting muscarinic antagonist; LRTA, leukotriene receptor antagonist; PR, pulmonary rehabilitation; Q, quartile; mMRC, modified British medical council dyspnoea scale; SABA, short-acting beta-agonist; SAMA, short-acting muscarinic antagonist; SD, standard deviation.

**Table 2:** Factors associated with the number of repetitions in the one-minute sit-to-stand test (1minSTS) in people with chronic obstructive pulmonary disease (COPD) over the 6-month period (n = 108).

(n = 108)	1minSTS
**Fixed effects (factors)**	**Estimate (95%CI)**
(Intercept)	**78.23 (63.55; 92.90)**
Sex (Female)	**−4.19 (−8.15; −0.23)**
Age	**−0.48 (−0.64; −0.32)**
BMI	**−0.51 (−0.84; −0.17)**
mMRC	**−2.89 (−4.24; −1.55)**
Time	**0.08 (0.03; 0.12)**
Sex (Female) * Time	0.02 (−2.57 × 10 ^−4^; 0.03)
Age * Time	**−8.89 ×** **10^−4^ (−1.61 ×** **10^−3^; −1.86 ×** **10^−4^)**
**Random Effects**	
σ^2^	9.84
τ_00_	52.90_Participant_
τ_11_	0.01_Participant.Time_
ρ_01_	0.47_Participant_
Observations	535
Marginal/Conditional R^2^	0.37/0.92

**Legend:** 1minSTS, one-minute sit-to-stand test; CI, confidence interval; BMI, body mass index; mMRC, modified British medical council dyspnoea scale; σ^2^, residual variance; τ, random effect standard deviation; ρ, correlation between intercept and slope; ICC, intraclass correlation coefficient; R^2^, coefficient of determination; * interaction with; in bold: *p*-value < 0.05.

## Data Availability

All data included in this study will be made available upon reasonable request to the corresponding author.

## Supplementary Materials

The following supporting information can be downloaded at: https://www.mdpi.com/article/10.3390/jcm11113052/s1, Figure S1: Number of repetitions in the one-minute sit-to-stand test (1minSTS) of participants with chronic obstructive pulmonary disease, over a period of 195 days. The 1minSTS values were adjusted for male subjects with an age of 65 years, a body mass index of 25, and a modified British medical re-search council scale score of 2. The linear mixed-effects model’s predicted values are represented by a solid line, 95% confidence prediction intervals by a light grey band, and observed values by dots.
