# Supplementary material for: Monthly Follow-Ups of Functional Status in People with COPD: A Longitudinal Study
Source: J Clin Med. 2022 May 28;11(11):3052. doi: 10.3390/jcm11113052 (PMC9181503; doi:10.3390/jcm11113052)
Supplement: Supplementary file 1 [file jcm-11-03052-s001.zip › jcm-1708131-supplementary.pdf]

Supplementary Material

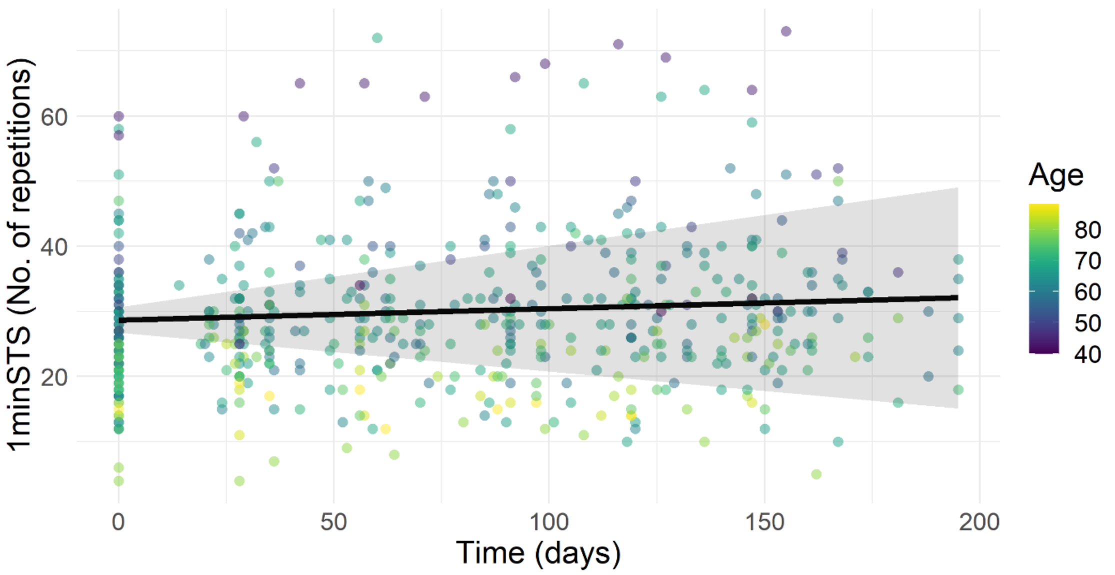

Figure S1. Number of repetitions in the one-minute sit-to-stand test (1minSTS) of participants with chronic obstructive pulmonary disease, over a period of 195 days. The 1minSTS values were adjusted for male subjects with an age of 65 years, a body mass index of 25, and a modified British medical research council scale score of 2. The linear mixed-effects model's predicted values are represented by a solid line, 95% confidence prediction intervals by a light grey band, and observed values by dots.
